# Chyluria after Partial Nephrectomy: Case Report and Review of the Literature

**DOI:** 10.1100/tsw.2009.5

**Published:** 2009-01-18

**Authors:** Ronald J. Kim, Fadi N. Joudi

**Affiliations:** Department of Urology, University of Iowa, Iowa City, IA, USA

**Keywords:** chyluria, partial nephrectomy

## Abstract

We report a case of 61-year-old male who presented with chyluria after partial nephrectomy. During workup for appendicitis, an incidental exophytic renal mass was revealed on CT scan. The patient ultimately underwent uncomplicated open partial nephrectomy. Postoperatively, his JP drain output turned milky white with urine remaining clear. JP fluid analysis was consistent with lymph. At 3 weeks postsurgery, his drain output decreased, but his urine turned milky white. Urinalysis confirmed fat in the urine. CT imaging revealed chyloma/urinoma with extravasation. The patient was initially treated conservatively, with a medium-chain fatty acid diet and then ureteral stenting. His stent was eventually removed and his chlyuria resolved 14 weeks later.

In nonendemic countries, nonparasitic chyluria is exceedingly rare and postsurgical chyluria even more so. We review the sequelae of untreated disease and surgical options for intractable chyluria not responsive to conservative management.In non-endemic countries, non-parasitic chyluria is exceedingly rare, and post surgical chyluria even more so. We review the sequelae of untreated disease and surgical options for intractable chyluria not responsive to conservative management.

## INTRODUCTION

Chyluria is a result of a lymphatic fistulous communication to the collecting system. Treatment and monitoring is important as sequelae of the disease include nutritional deficiency, recurrent clot colic, UTI, hematuria, and, in extreme cases, immunosuppression (lymphocytopenia and IgA deficiency)[1]. Typically, in endemic areas, chyluria is due to lymphatic stasis and obstruction due to infection by parasitic filarial roundworms of the genera *Wuchereria*, *Brugia*, and *Onchocerca*. Nonparasitic causes include tuberculosis, infection/abscess, and pregnancy, and it can be secondary to neoplasms or iatrogenic (postsurgical). The incidence after kidney surgery is unknown as only a few published case reports exist.

## CASE REPORT

A 61-year-old white male initially presented to our institution's emergency treatment center with right lower quadrant pain clinically consistent with appendicitis. CT scan was consistent with acute appendicitis, but also showed an exophytic 5.3- × 3.7- × 4.8-cm solid enhancing mass. After uncomplicated laparoscopic appendectomy, the remainder of his staging (chest radiograph and liver enzymes) was negative. Due to the exophytic nature, location, and size of the mass, the patient underwent open left lower pole partial nephrectomy approximately 6 weeks after his appendectomy.

Intraoperatively, the hilum was dissected, and the renal artery and vein were exposed. The hilum was not clamped as it was felt that the mass could be excised without clamping, thus avoiding ischemia. Lymph node dissection was not performed. The collecting system was violated and closed with a 4-0 running vicryl suture. The renal parenchyma was coagulated with an argon beam. Surgicel bolsters (Ethicon Endo-Surgery, Cincinnati, OH) were placed in the tumor bed to fill the defect and the parenchyma was approximated using 0 vicryl sutures in an interrupted fashion. Floseal (Baxter, Deerfield, IL) and Bioglue (Cryolife, Inc., Kennesaw, GA) were then applied over the bolsters. A Jackson-Pratt (JP) drain was left to gravity drainage.

Final pathology revealed renal cell carcinoma, clear cell type, Fuhrman grade 2 with negative margins. On his final hospital day, JP drain output was milky appearing and fluid analysis revealed creatinine of 0.7 mg/dl and a triglyceride level of 1966 mg/ml. A chylous leak was suspected and the patient was discharged on postoperative day 4 on a medium-chain fatty acid diet with the JP drain in place.

At 3 weeks postprocedure, the patient reported that the JP output continued to be milky appearing, but had recently become scant, and that his urine appeared cloudy/milky for the first time. A CT scan at that time revealed a small perinephric fluid collection and small area of urine extravasation. We elected to insert a double-J stent. Another follow-up CT scan 3 weeks later showed a decrease in size of the fluid collection and no evidence of extravasation. The patient reported no clinical symptoms, as well as increasing episodes of clear urine with only intermittent milky-appearing urine. There was still fat present in his urine on laboratory examination. His serum albumin was within normal range. Due to his improvement, the stent was left in place.

Six weeks later, the patient reported that his urine was only slightly whitish after meals, but normal at other times. Due to his improvement and because he was entirely asymptomatic throughout this time, his stent was removed. Approximately 8 weeks later, his milky urine had completely resolved.

## DISCUSSION

In endemic countries, there is an abundance of literature on the various treatments of persistent chyluria. Medical management is limited to medium-chain fatty acid diets (avoidance of long-chain triglyceride) and coconut oil (which is high in medium-chain fatty acids)[2]. Surgical management includes endoscopic sclerotherapy[3] and renal pedicle lymphatic disconnection[4]. Agents that have been used for sclerotherapy include silver nitrate, N-butyl-2-cyanoacrylate[5], povidone iodine, and even radiographic contrast media. Cure rates of 59–82% have been published, but late recurrences up to 50% have also been reported with sclerotherapy[6].

The largest series of open surgical lymphorenal disconnection consisted of 383 patients and reported a cure rate of 80–90% and a 25% recurrence rate of those followed to 2 years[6]. Renal pedicle stripping has been reported laparoscopically, both by transperitoneal and retroperitoneal approaches[2]. Retroperitoneal lymphatic disconnection includes nephrolympholysis, ureterolympholysis, hilar vessel stripping, fasciectomy (Gerota's), and nephropexy. Case series report good results, but the series are relatively small in number[2,4]. Renal autotransplantation has also been reported after initial failure with renal pedicle stripping[7].

Nonparasitic chyluria after renal surgery is rare and limited to a few case reports in the literature. Tuck et al. reported chyluria after radical nephrectomy where a fistulous connection developed to the distal half of the ureter left *in situ*[5]. This was treated with endoscopic injection of povidone iodine and then with N-butyl-2-cyanoacrylate. Thrasher and Snyder reported chyluria after percutaneous nephrolithotomy, which resolved with total parenteral nutrition and no other surgical intervention[8]. Miller et al. reported four cases of chyluria after partial nephrectomy; it is noteworthy that none of these patients were diagnosed clinically, but only after CT diagnosis of fat-fluid level in the bladder, suggesting that subclinical chyluria may be more common than previously thought[9]. Only two of these four patients noted occasional cloudy urine and none had intervention due to their asymptomatic nature.

Renal capsule and parenchymal lymphatics drain to the renal hilum on their way to interaortocaval nodes or lateral aortic nodes, depending on laterality[10]. Chylous ascites has been reported much more frequently after nephrectomy than chyluria. A recent review noted 34 cases of chylous ascites in the English literature after nephrectomy for various reasons[11]. Chyluria after nephrectomy is extremely rare and only one documented case report can be found in the English literature. It requires the formation of a fistulous connection from the transected hilar vessels to the remaining urinary system, either to the ureteral stump or the bladder itself. The review of the literature above implies that chyluria is more common after nonradical renal surgery. The renal capsule and cortex are supplied with generous lymphatic drainage with an abundance of lymph vessels[12]. A fistulous connection from the capsule or cortex to the collecting system logically would be a more common event, especially after partial nephrectomy with violation into the collecting system.

In our case, a chylous leak developed and was draining through the JP drain. Once the JP was removed, a communication with the collecting system developed and, thus, the chyluria. At the time of surgery, multiple modalities (Surgicel, Floseal, Bioglue) were used to prevent urine leakage and chyluria, the main one being closure of the renal pelvis with a running suture. Argon beam coagulation also should have closed any lymphatic vessels violated after the mass was excised. The kidney is known to have extremely rich lymphatics in the capsule[12] and whether incomplete ablation of these with the argon beam led to chylous leak is speculative. Regardless of the etiology in this case, intraoperative control of any visible lymphatic vessels and the use of sealants can potentially help to avoid this complication.

Conservative treatment with a medium-chain fatty acid diet did not promote early closure of the fistula. In this case, it took 14 weeks for the chylous leak to resolve. Although our patient noted clinically significant signs of chyluria, his albumin remained normal and he remained asymptomatic. Stent insertion was performed to alleviate urine leakage, but it is not clear if this promoted healing of the chylous fistula itself. Regardless, this case demonstrates that chyluria can be a complication of partial nephrectomy and initial conservative management in an asymptomatic patient with iatrogenic chyluria is prudent, as these can resolve spontaneously. The patient can be reassured, and invasive interventions like sclerotherapy and operative measures should be avoided unless the patient is affected from the sequalae of chyluria. This is a postoperative complication like many others where all the patient needs is a "tincture of time".
